# Increase in Strength and Fretting Resistance of Alloy 718 Using the Surface Modification Process

**DOI:** 10.3390/ma11081366

**Published:** 2018-08-06

**Authors:** Auezhan Amanov, Rakhmatjon Umarov, Tileubay Amanov

**Affiliations:** 1 Department of Mechanical Engineering, Sun Moon University, Asan 31460, Korea; umarov92@inbox.ru; 2 Institute of Mechanics and Seismic Stability of Structures, Academy of Sciences, Tashkent 100125, Uzbekistan; amanov43@mail.ru

**Keywords:** Alloy 718, surface hardness, surface residual stress, grain size, fretting failure, corrosion

## Abstract

This work comparatively investigated the strength (hardness, yield strength, dynamic elastic modulus, and surface residual stress), fretting failure, and corrosion resistance of the as-received and treated Ni-based superalloy Alloy 718. The goal of the current research is to improve the hardness, fretting wear, and corrosion resistances of Alloy 718 through the ultrasonic nanocrystal surface modification (UNSM) process with the aim of extending the lifespan of aircraft and nuclear components made of Alloy 718. The experimental results revealed that the surface hardness increased by about 32%, the fretting wear resistance increased by about 14%, and the corrosion resistance increased by about 18% after UNSM process. In addition, the UNSM process induced a tremendous high compressive surface residual stress of about −1324 MPa that led to an increase in yield strength and dynamic Young’s modulus by about 14 and 9%, respectively. Grain size refinement up to ~50 nm after the UNSM process is found to be responsible for the increase in surface hardness as well. The depth of the effective layer generated by the UNSM process was about 20 µm. It was concluded that the UNSM process played a vital role in increasing the strength and enhancing the corrosion and fretting resistances of Alloy 718.

## 1. Introduction

Alloy 718 is mainly used in aerospace and nuclear applications in the range of operating temperature of 650–675 °C because of relatively excellent mechanical properties, fusion weldability, good, excellent oxidation resistance, corrosion resistance, and creep at elevated temperatures [1]. However, the relatively poor fretting wear resistance of Alloy 718 may restrict its use without any surface engineering [2]. Alloy 718 usually exhibits poor wear resistance, resulting in shortening the service life of components owing to the relatively low hardness in spite of its excellent properties such as corrosion, erosion, oxidation, etc. [3]. One of the main issues in the aerospace and nuclear industries is fretting damage, which can be derived from oscillating motion, and it may lead to high nominal stress between two fretting bodies, resulting in high wear or even fatigue limit [4]. Alloy 718 can be hardened by the presence of precipitation of *γ*′ (Ni_3_(Al,Ti)) and *γ*″ (Ni_3_Nb) phases within a (face centered cubic) FCC structure. The latter phase can be transferred into Ni_3_Nb—*δ* one at such a high temperature, where a softening can take place [5]. Also, *δ* phase (Ni_3_Nb) is a vital phase, which precipitated in the range of 750~1020 °C [6,7]. In general, in Alloy 718, the precipitation of *δ* phase does not provide any strengthening mechanism, but it controls the microstructure [8]. It is well established that Alloy 718 can be directly used for various structural applications with no heat treatment thanks to the standard precipitation treatment leading to an increase in strength [9,10]. Lin et al. have reported earlier that a high amount of *δ* phase significantly decreased the strength and plasticity of Alloy 718 [7]. On the other hand, the balanced amount of *δ* phase may serve to alter the microstructure in terms of grain size refinement and dislocation impediment that led to an increase in strength [11]. However, the enhancement in strength of Alloy 718 by the presence of *δ* phase was not good enough to be used in real applications. Moreover, it is well known that the strength of Alloy 718 can be also substantially improved by various heat treatment processes. For instance, Chlebus et al. have investigated the heat treatment response to the microstructure and mechanical properties of Alloy 718 produced by additive manufacturing process [12]. In total, four series of heat treatments were performed at various annealing temperatures. It was found that the highest hardness of Alloy increased from 312 to 461 HV. Another study also claimed that the mechanical properties of Alloy 718 can be increased by annealing and hot isostatic pressing (HIP) + annealing processes, resulting in an increase in hardness by about 10 and 31%, respectively [13]. Raghavan et al. have also investigated the effect of heat treatment on mechanical properties of Alloy 718 [14]. The results revealed that increase in homogenization temperature coarsened the grain size that led to softening, but the strength increased remarkably after ageing treatment due to the precipitation hardening of Alloy 718.

In this regard, a number of investigations have been performed to increase the strength of Alloy 718 [15,16,17]. For instance, Zheng et al. have studied the influence of carbon (C) content on control in strength of Alloy 718 [18]. It was found that the amount of precipitation was reduced, and the grain size of carbide was refined with decreasing C content, leading to an increase in strength. Liu et al. have tried to control the mechanical properties of Alloy 718 using the electromagnetic stirring [EMS] process [19]. Interestingly, a tensile strength of Alloy 718 subjected to EMS process was found to be higher in comparison with that of the unprocessed Alloy 718 because of the increased surface hardness and induced compressive residual stress. In addition, Farber et al. have found the relationship between the mechanical properties and microstructure of four different combinations of Alloy 718: solution and ageing heat treatment, heat treatment along with hot isostatic pressing, heat treatment along with shot peening, and heat treatment together with hot isostatic pressing and shot peening. The results showed that even though the heat treated specimen exhibited better results in terms of surface hardness, the specimen subjected to heat treatment and hot isostatic pressing revealed similar results in terms of tensile strength and ductility, in which the surface hardness was found to be much lower compared to that of the heat treated one [8]. Surface modification methods such as shot peening and laser shock peening have also been widely used to increase the mechanical properties of Alloy 718. For example, Chamanfar et al. have used a shot peening process to increase the mechanical properties of Alloy 718 [20]. Gill et al. have analyzed the effect of laser shock peening on the hardness and residual stress [21]. Both surface modification methods increased the mechanical properties due to the severe plastic deformation of the surface as a result of inducing high strain and the transformation of tensile residual stress into compressive one. 

In this current paper, Alloy 718 was treated by a relatively new ultrasonic nanocrystalline surface modification (UNSM) process to achieve high strength and excellent resistances to fretting and corrosion simultaneously. The main goal of the current research is to evaluate the impact of UNSM process in terms of surface roughness, surface hardness, surface residual stress, grain size, etc., on the increase in strength, fretting wear, and corrosion resistance that lead to an increase in lifespan of various components and parts made of Alloy 718 in both aerospace and nuclear industries. The effective depth of UNSM process was confirmed by cross-sectional observation. The impact of the UNSM process on the surface hardness and tensile behavior was obtained using hardness and tension tests, while the corrosion resistance was studied using the potentiodynamic polarization test method. Finally, the level of fretting failure was investigated by fretting wear tester under dry conditions.

## 2. Materials and Methods

### 2.1. Material

In this study, a number of wrought specimens made of solution-annealed Alloy 718 were used. The specimens were melted at VDM Metals (USA/FP), precipitation heat treated at 720 °C, and then air cooled. The grain size of specimens determined in accordance with a standard [22]. ASTM-E-112-13. The mechanical properties and chemical composition of Alloy 718 provided by the supplier are listed in Table 1 and Table 2, respectively. Before and after UNSM process, all the specimens were washed in acetone ((CH_3_)_2_CO) for 5 min. Then, the optimized UNSM parameters listed in Table 3 were selected to be applied to Alloy 718. Detailed information about the UNSM process and its parameters are available elsewhere [23,24].

### 2.2. Characterization

Before measuring the surface roughness and surface hardness, the specimens were ultrasonically cleaned in distilled water (H_2_O) for 10 min to keep the surface clean. The average surface roughness (*R_a_*) of the specimens was obtained using a surface profilometer (SJ-210, Mitutoyo, Kawasaki, Japan) with a scanning length 4 mm at a scan rate of 10 mm/min. The surface hardness was measured at a load of 300 gf for 10 s by micro-Vickers hardness tester (MVK E3, Mitutoyo, Japan). The indent points were randomly selected at a distance of 300 µm away from each other. The indent size was about 40–45 µm. The surface roughness and surface hardness measurements were repeated three times to give a good statistical representation of the results. To observe the difference in microstructure, the specimens were embedded on resin, and the conductive resin used to embed the metal sample was produced by press. The top surface of the specimens was mechanically polished using a silicon carbide (SiC) sandpaper with a grit in the range of 400 to 2000. The specimens were electrolytically etched in standard nital etchant solution (15% nitric acid and 85% ethanol) at 6 V for 10 s using an electropolisher-etcher (ElectroMetTM4, Buehler, Lake Bluff, IL, USA) to reveal the change in microstructure after UNSM process.

**Figure 1:**
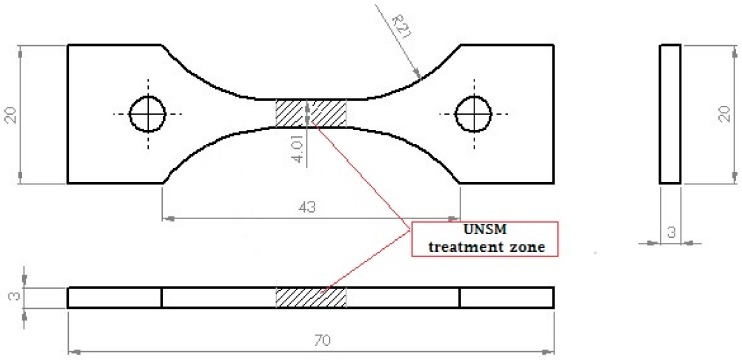
A schematic view of a tensile test specimen.

The mechanical properties were obtained using a tensile tester (Zwick/Roell Z010, Borchen, Germany) with increasing load from 0 to 10 kN at a loading rate of 5 mm/min. The dimensions of the tensile specimen are shown in Figure 1. The dynamic elastic modulus was measured with respect to temperature in the range of 100 to 400 °C by a resonant frequency. The temperature was controlled using an induction heating system with a medium frequency of 100 Hz. Tensile and dynamic Young’s modulus tests were also repeated at least three times. Detailed information on measurement of dynamic Young’s modulus is available elsewhere [25].

The fretting wear resistance was assessed by an Optimol SRV IV (Munich, Germany) tester at room temperature under dry conditions against SAE 52100 bearing steel ball with a diameter of 10 mm. The fretting wear test conditions are listed in Table 4.

The corrosion resistance was evaluated by a potentiodynamic polarization tester (Solartron SI 1287, Gainesville, GA, USA) with three electrodes as a saturated calomel electrode (SCE), and platinum mesh, and specimen as reference. At a scanrate of 1 mV/s with a scan range of −0.5 V to +1.5 V in 3.5% NaCl solution corrosion resistance of as-received and UNSM-treated specimens was measured at room temperature. The exposed area of the specimen was 105.6 mm^2^.

X-ray diffraction (XRD) pattern using a sin^2^ψ method with a scanning speed 1/min and surface residual stress were obtained by an X-ray diffractometer (Bruker D8 Advance, Karlsruhe, Germany). The measurement area was 300 µm in diameter, and the penetration depth was 500 µm. The fretting wear rate was calculated using a fretting wear scar profile obtained by a laser scanning microscopy (VK-X100 Series, Keyence, Osaka, Japan). The effective depth of UNSM process was observed by field-emission (FE)-SEM images. It needs to be mentioned here that all the experimental tests and measurements were repeated three times to give a good statistical representation of the results.

## 3. Results and Discussion

### 3.1. Surface Roughness and Hardness

**Figure 2:**
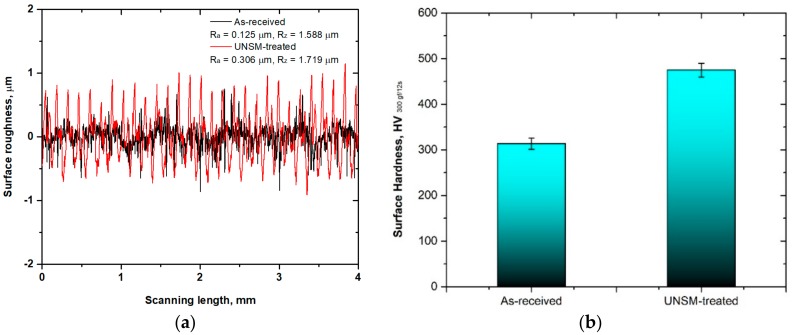
Surface roughness profile (**a**) and hardness (**b**) of the as-received and UNSM-treated specimens.

The surface roughness profile and surface hardness of the as-received and UNSM-treated specimens are shown in Figure 2. In fretting conditions, it is essential to have a rough surface; therefore, the average surface roughness (*R_a_*) of the UNSM-treated specimen was intentionally modified to be in the range from 0.25 to 0.32 µm by creating high peaks and valleys, as illustrated in Figure 2a. It has previously been reported that a rougher surface can better avoid the initiation of fretting failure in comparison with the smoother one [26]. Meanwhile, it is important to mention that the UNSM process is also used as a surface smoothing process that achieves a very smooth surface with a surface roughness *R_a_* of several nanometers [27]. In addition to surface roughness, the performance of the UNSM process on the surface hardness, which is an indication of resistance to deformation, was also evaluated as illustrated in Figure 2b. Obviously, the UNSM-treated specimen exhibited a higher surface hardness of 456 HV in comparison with the as-received one of 314 HV. The Hall-Petch expression explains the enhanced surface hardness of the UNSM-treated specimen, in which the surface hardness can be controlled by refining the coarse grain size [28]. It is well established that the UNSM process generates a nanocrystal layer up to a certain depth from the top surface, along with refined nano-grain size down to ~50 nm [29]. Moreover, an increase in surface hardness may be attributed to the work hardening by UNSM process as a result of the generated surface and subsurface severe plastic deformation (SPD). Alloy 718 can be also hardened by the presence of precipitation of *γ*′ (Ni_3_(Al,Ti)) and *γ*″ (Ni_3_Nb) phases. The refined nano-grain size as a function of depth from the surface of Alloy 718 was reported previously, in which the finest grain of the top surface was about 30 nm [30].

### 3.2. Tensile and Dynamic Elastic Modulus

**Figure 3:**
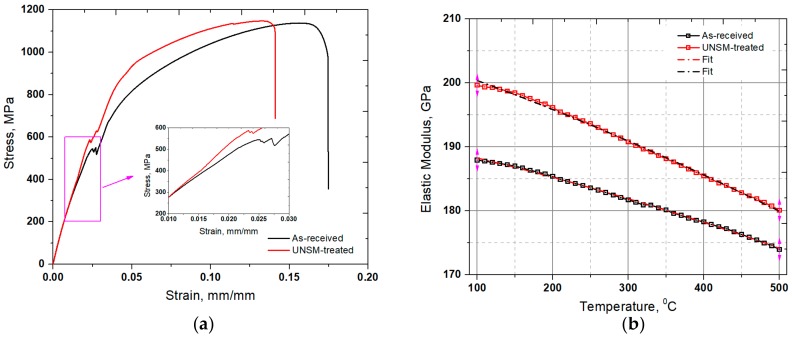
Stress-strain curve (**a**) and elastic modulus (**b**) change of the as-received and UNSM-treated specimens.

The mechanical properties of the as-received and UNSM-treated specimens were evaluated by a tensile test, whereas the typical stress-strain curves are plotted in Figure 3a. It is clear that there is no significant difference in ultimate tensile strength (UTS) after the UNSM process, but the yield strength (YS) increased by about 14%. Unfortunately, the UNSM process lost the ductility of the as-received specimen by about 20%. The relationship between strength vs. ductility of engineering materials is still an important issue in Materials Science. Lu has suggested that a gradient nanostructured material can improve both the strength and ductility compared to the nanostructured one [31]. Hence, it is suggested to generate a gradient nanocrystal structure using the UNSM process, which may lead to a further increase in both the strength and ductility [32]. Metallic materials are ductile, and they have a relatively lower strength but a relatively higher toughness or energy to failure because of greater ductility/post yield deformation. Therefore, a gradient nanostructured Alloy 718 is needed to control the strength vs. ductility. In addition, dynamic elastic modulus, which is the ratio of stress to strain under vibratory conditions, as a function of temperature of the specimens, is presented in Figure 3b. The results showed that the dynamic elastic moduli of the as-received and UNSM-treated specimens were 187.24 and 199.79 GPa at a temperature of 100 °C, respectively. By increasing the temperature, the dynamic elastic modulus of the UNSM-treated specimen was higher throughout the range of temperatures compared to the as-received one, in which the amount of increase was reduced at higher temperature, as depicted in Figure 3b.

### 3.3. Surface Residual Stress and XRD Pattern

**Figure 4:**
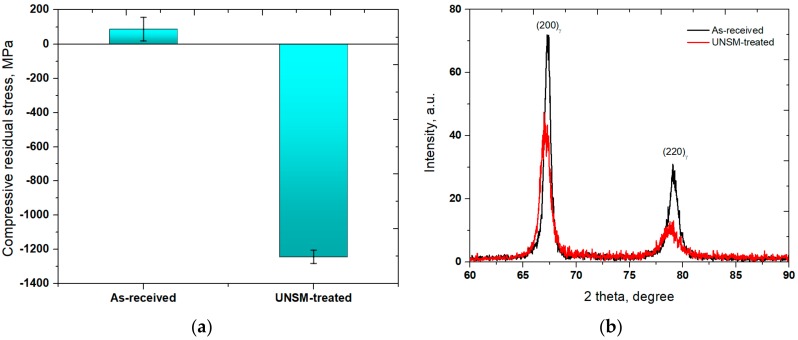
The surface residual stress measurement results (**a**) and XRD patterns (**b**) of the as-received and UNSM-treated specimens.

Figure 4a presents the surface residual stress measurement results obtained from the as-received and UNSM-treated specimens. The surface residual stress of the as-received specimen was found to be 0.178 GPa, which changed after the UNSM process to a value of about −1.324 GPa. One of the main influential factors in the ductility and fatigue of Alloy 718 is the compressive surface residual stress owing to detain crack initiation and propagation [33]. An XRD was utilized to analyze the grain size, micro-strain, and phase transformation alteration of the as-received and UNSM-treated specimens. In Figure 4b, it can be seen clearly that the intensity of both diffraction peaks of <200> and <220> reduced significantly, whereas the full width at half maximum (FWHM) widened after UNSM process. The FWHM of the primary <200> peak was found to be 0.49 and 0.61 for the as-received and UNSM-treated specimens, respectively. The grain size refinement played an important role in reducing the intensity and increasing the FWHM value, and also in increasing the dislocation density [34].

### 3.4. Cross-Sectional Observation by SEM

**Figure 5:**
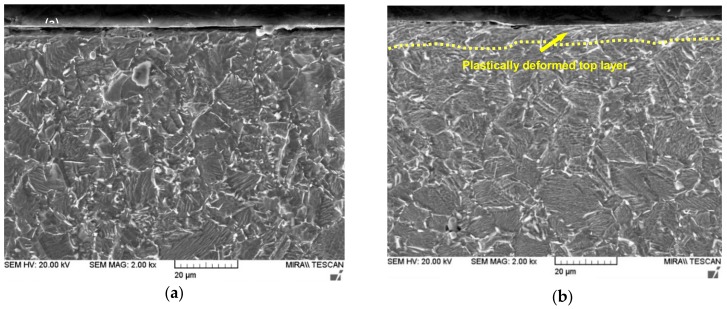
Cross-sectional SEM images of the as-received (**a**) and UNSM-treated (**b**) specimens.

The cross-sectional SEM images of the as-received and UNSM-treated specimens are presented in Figure 5. It is obvious that the plastically deformed top surface with a thickness about 10 µm of the specimen after the UNSM process can be observed in Figure 5b. It can be also seen that the deformation occurred along the UNSM treatment direction, as indicated by yellow arrow. In general, once a material is subjected to SPD process, an increase in grain boundaries and refinement in grain size take place. It has been reported earlier that the UNSM process was able to refine grain size up to ~50 nm [29]. Several mechanisms of strengthening of Alloy 718 are available in the literature, in which grain size refinement is one of the possible mechanisms with which to increase strength [35]. It is announced earlier that the refined grain size using SPD methods less than 10 nm was found to be detrimental [36]. Also, slip or dislocation may occur along the grain boundary during SPD, in which the increased grain boundaries may block dislocation motion due to the orientations of neighboring grains. Hence, a nano-grained material has much higher hardness compared to coarse grained one because of the higher number of grain boundaries in nano-grained materials.

### 3.5. Friction Coefficient and Fretting Wear Resistance

**Figure 6:**
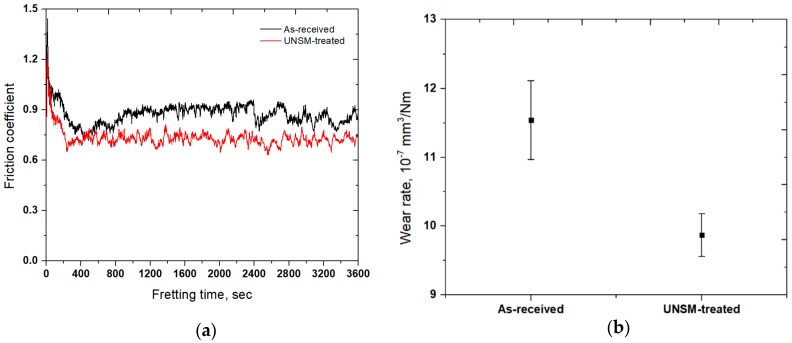
Friction coefficient (**a**) and fretting wear resistance (**b**) of the as-received and UNSM-treated specimens.

The friction coefficient as a function of fretting time of the specimens is shown in Figure 6a, in which the friction coefficient increased drastically at the start point of the test and then reduced gradually for the first 10–14 min, and it got stabilized throughout the testing time. In other words, the average friction coefficient of the as-received specimen reduced from 0.88 to 0.72, corresponding to 18% after the UNSM process. A high friction coefficient of the specimens at the start point of the test is attributed to the initial asperity contact that increases contact pressure significantly, since only a small number of asperities came into contact at the interface. Moreover, Figure 6b shows the evaluation of the fretting wear resistance based on the fretting wear scar profiles. It was revealed that the UNSM process reduced the fretting wear resistance significantly. The main reason for the reduction in the friction coefficient of the UNSM-treated specimen is the initial surface roughness, which reduced the true contact area between the surfaces of the specimen and the ball. It is worth mentioning here that high peaks and valley of rough surface may serve as additional space at the contact interface for wear debris formed during fretting oscillation. The improvement in wear resistance of the specimen subjected to UNSM process is due to the hardening and alteration of coarse grained structure into nano-grained one. Recently, the role of the UNSM process on the control of friction and fretting wear resistance of Inconel 690 alloy at 25 and 80 °C was reported in our previous publication [19]. It was reported that the friction behavior and fretting wear resistance of the UNSM-treated specimens were improved at both temperatures compared to the as-received ones. Another application of UNSM process on AlCrN coating with the aim of increasing the fretting resistance was reported earlier [37]. Interestingly, the UNSM process was capable of reducing the friction coefficient, which led to an enhanced fretting wear resistance.

**Figure 7:**
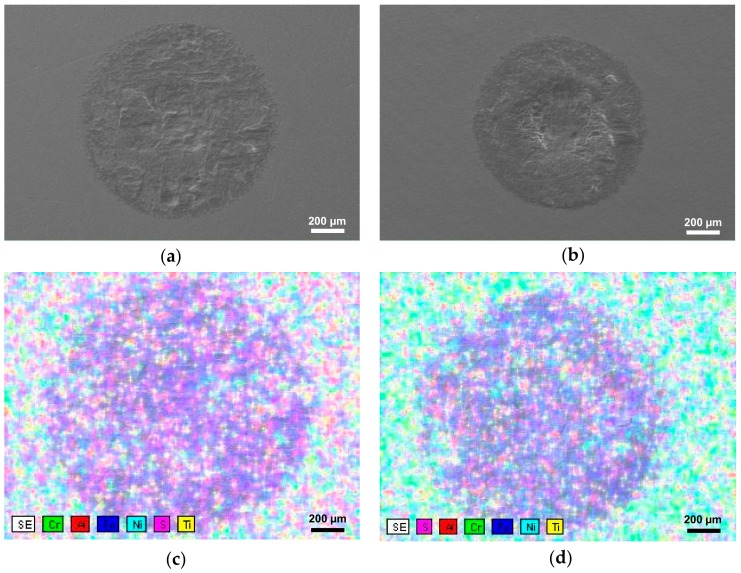

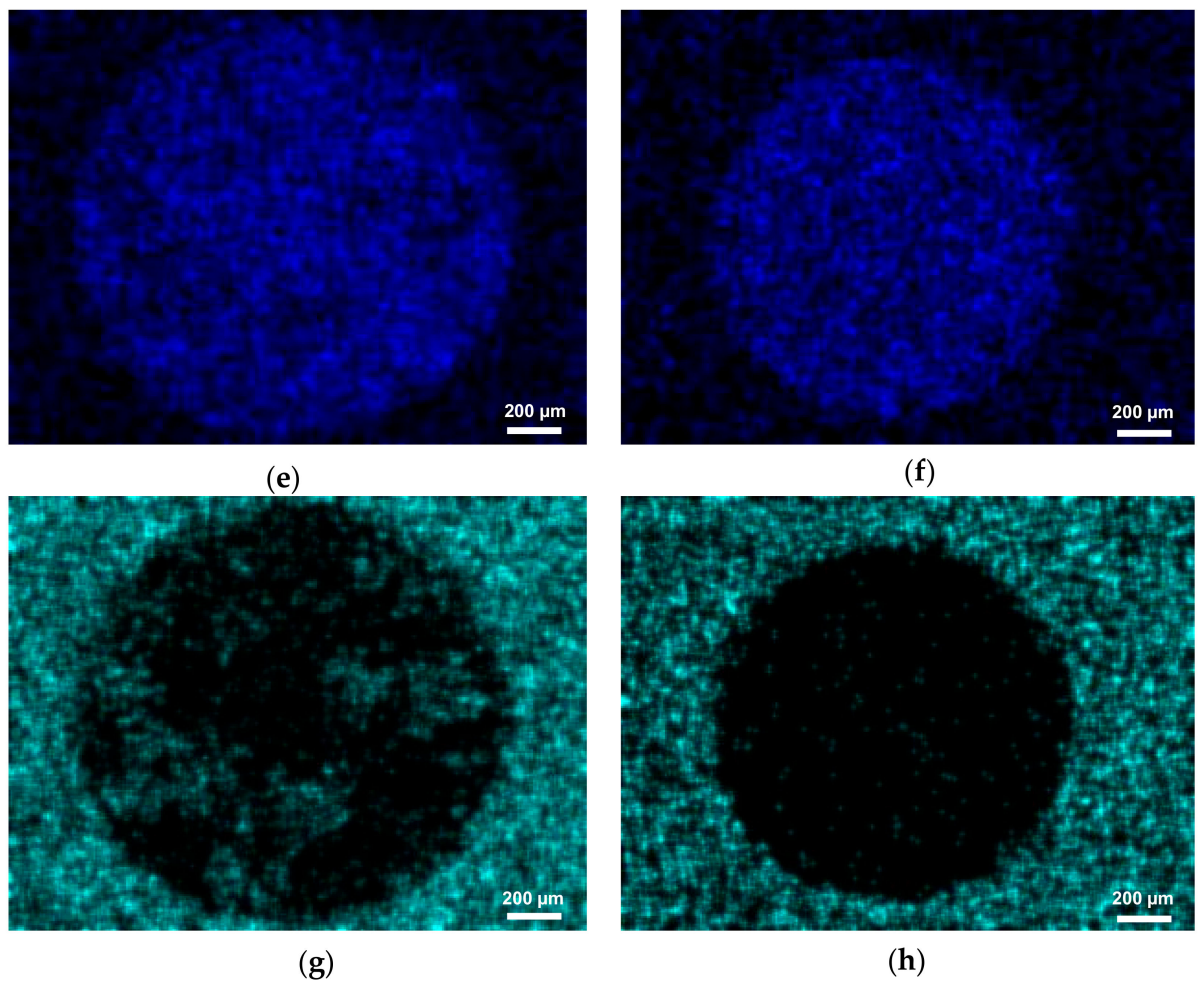
SEM images of the fretting wear scar formed on the surface of the as-received (**a**) and UNSM-treated (**b**) specimens. The composition mapping image of the fretting wear scar on the surface of the as-received (**c**) and UNSM-treated (**d**) specimens. Distribution of Fe (**e**,**f**), and Ni (**g**,**h**) over the fretting wear scar formed on the surface of the as-received (**a**) and UNSM-treated (**b**) specimens, respectively.

The fretting wear scars formed on the surface of the specimens were characterized by SEM as presented in Figure 7a,b. It was found based on the SEM images that the diameter of fretting wear scar of the as-received and UNSM-treated specimens was about 1.202 and 1.048 mm in diameter, respectively. Besides the diameter of fretting wear scar, it is of interest to investigate the chemistry and wear mechanisms of fretting wear as well. Figure 7c,d presents the chemical mapping of the fretting wear scar. Basically, as presented in Figure 7e,f, the fretting wear scar was covered with Fe, which was transferred from the counterface. The EDS spectroscopy revealed that Ni composition in the fretting wear scar on the UNSM-treated specimen was found to be smaller than the as-received one as shown in Figure 7g,h due to the high amount of Fe transferred from the counter ball. Hence, the application of UNSM process was beneficial to controlling the friction behavior and the fretting wear resistance.

### 3.6. Corrosion Resistance

**Figure 8:**
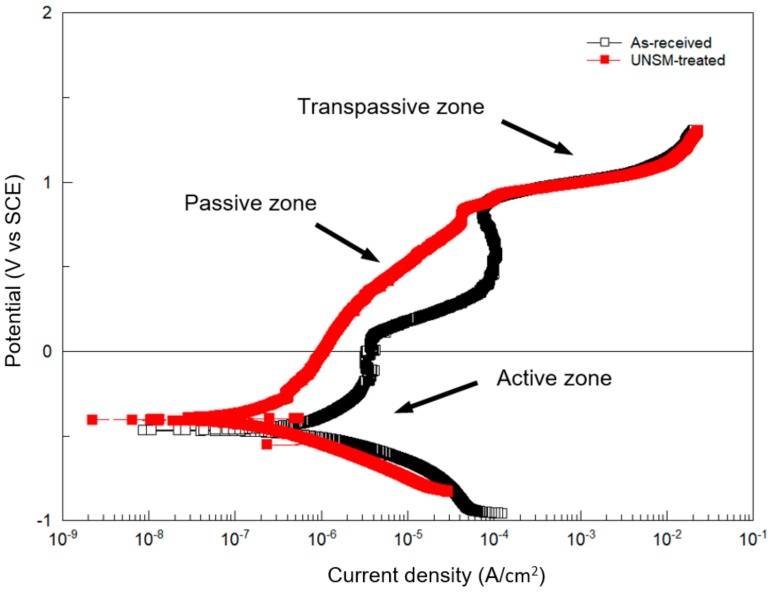
Potentiodynamic polarization curves of the as-received and UNSM-treated specimens.

Electrochemical tests were performed to explore the effect of the UNSM process on the corrosion resistance of Alloy 718. The comparison of Tafel curves for the as-received and UNSM-treated specimens in 3.5% NaCl solution is presented in Figure 8. The corrosion potential and breakdown of Alloy 718 are −0.45 and 0.90 V_SCE_, respectively. Clearly, it can be seen that the curves are divided into three zones as indicated by arrows. After UNSM process, the corrosion potential increased (*E_corr_*), and the corrosion current density (*i_corr_*) decreased in comparison with the as-received specimen, as shown in Figure 8. It is evident that the corrosion potential (*E_corr_*) after the UNSM process shifted to a noble direction, while the corrosion current density (*i_corr_*) shifted to the left side, corresponding to a higher resistance to corrosion compared than the as-received one. More negative corrosion potential and high corrosion current are indicative of lower resistance to corrosion. In other words, the smaller values of the corrosion current density indicate higher corrosion resistance, and the more positive the values of the corrosion potential are, the higher is the corrosion resistance [38]. Identified transpassive zone of the as-received specimen, as shown in Figure 8, could be probably due to anodic reaction.

The values of electrochemical test results are listed in Table 5. It was found that the corrosion potential and current density of the UNSM-treated specimen improved due to the inception of a dissolution on the anode, and galvanic coupling of cathodic and anodic area was sped up on the UNSM-treated specimen. The magnitude of corrosion potential is not the only property evaluating the corrosion resistance of Alloy 718. In general, the specimens with negative potential tend to have a lower resistance to corrosion. However, accelerated passivation inhibited the cathodic reaction, in which the motion of potential to negative corrosion takes place. No significant difference in χ^2^ (chi-square) deviation was found, as shown in Table 5, in which the low values of the associated χ^2^ indicate that the circuit was able to fit the experimental data accurately. In other words, the small values of chi-square indicates a better fit. The obtained results imply that the corrosion resistance of the UNSM-treated specimen increased compared to the as-received one. Lee et al. have studied the influence of shot peening and UNSM processes on the corrosion resistance of AISI304 stainless steel [39]. It was revealed that the UNSM-treated specimen with a smoother surface showed better corrosion resistance in comparison with the as-received and shot peened specimens. However, Hou et al. systematically investigated the influence of UNSM process on the corrosion behavior of AZ31B Mg alloy [40]. No improvement in corrosion behavior after UNSM process was found due to the increase in dislocation density induced by UNSM process. Reduction in surface roughness and change in microstructure after UNSM process can play an important role in increasing and decreasing the corrosion resistance. Hence, it is worth mentioning here that a rough surface roughness was prepared intentionally by UNSM process to improve the fretting wear resistance so that a rough surface could increase the corrosion resistance. Furthermore, Miyamoto overviewed the corrosion behavior of nano-grained materials using SDP methods and reported that there are some contradictory results [41]. Most of the reviewed papers reported that the refined grain size may increase the corrosion resistance following Ralston’s rule [42].

## 4. Conclusions

The influence of UNSM process on the strength; hardness; tensile strength; dynamic elastic modulus; and surface residual stress, fretting, and corrosion resistances was investigated. It was found that the average surface roughness and the surface hardness were increased by about 58% and 27%, respectively. XRD diffraction pattern intensity and FWHM were reduced and broadened after the UNSM process, resulting in grain size refinement and generating a high surface compressive residual stress. Both the friction coefficient and the fretting wear were improved after the UNSM process. The corrosion resistance was also increased slightly after UNSM process. It was learned that an optimization of the UNSM process parameters is in need to further increase the resistance to corrosion. Accordingly, it can be summarized that the UNSM process played a vital role in increasing the strength, corrosion, and fretting resistances of Alloy 718 that are beneficial to extending the service lifespan of aircraft and nuclear components.

## Figures and Tables

**Table 1:** Mechanical properties of Alloy 718.

Tensile Strength, MPa	Yield Strength, MPa	Elastic Modulus, GPa	Shear Modulus, GPa	Poisson’s Ratio	Elongation, %
1150	950	211	77.2	0.294	25

**Table 2:** Chemical composition of Alloy 718 in wt %.

Fe	Cr	C	Ti	Mn	Si	Ni	S	P	Mo	Nb	Al
17.62	18.84	0.024	0.95	0.02	0.06	53.64	0.002	0.003	3.08	5.23	0.53

**Table 3:** UNSM process parameters.

Frequency, kHz	Amplitude, µm	Speed, mm/min	Load, N	Feed-Rate, µm	Ball Diameter, mm	Ball Material
20	50	2000	50	70	2.38	WC

**Table 4:** Fretting wear test conditions.

Frequency, Hz	Displacement Amplitude, µm	Normal Load, N	Test Duration, min	Hertzian Contact Stress, GPa
30	50	50	60	1.06

**Table 5:** Electrochemical test results derived from polarization curves in 3.5% NaCl solution.

Specimens	*E_corr_,* V	*I_corr_,* A/cm^2^	χ^2^
As-received	−0.48244	2.2734 × 10^−6^	8.439
UNSM-treated	−0.40361	1.1227 × 10^−6^	9.864
